# Evaluating the Suitability of Hazelnut Skin as a Feed Ingredient in the Diet of Dairy Cows

**DOI:** 10.3390/ani10091653

**Published:** 2020-09-14

**Authors:** Manuela Renna, Carola Lussiana, Vanda Malfatto, Mathieu Gerbelle, Germano Turille, Claudio Medana, Daniela Ghirardello, Antonio Mimosi, Paolo Cornale

**Affiliations:** 1 Department of Veterinary Sciences, University of Turin, Largo Paolo Braccini, 2, 10095 Grugliasco, Turin, Italy; 2 Department of Agricultural, Forest and Food Sciences, University of Turin, Largo Paolo Braccini 2, 10095 Grugliasco, Turin, Italy; carola.lussiana@unito.it (C.L.); vanda.malfatto@unito.it (V.M.); daniela.ghirardello@unito.it (D.G.); antonio.mimosi@unito.it (A.M.); paolo.cornale@unito.it (P.C.); 3 Institut Agricole Régional, 11100 Aosta, Italy; gerbellemathieu@gmail.com (M.G.); zootecnia@iaraosta.it (G.T.); 4 Department of Molecular Biotechnology and Health Sciences, University of Turin, via Nizza 52, 10125 Turin, Italy; claudio.medana@unito.it

**Keywords:** bovine milk, agro-industrial by-products, *Corylus avellana* (L.), polyphenols, fatty acids, vitamin E, feed efficiency, food-feed competition

## Abstract

**Simple Summary:**

Agriculture is estimated to generate about 700 million tons of waste annually in the Eurpoean Union (EU). Most of the by-products derived from the agricultural industry become organic waste, thus resulting in the loss of valuable nutrients and bioactive compounds and causing environmental and economic issues. Increasing the efficiency of waste management is a major global challenge that must be met in order to minimize the abovementioned negative impacts. Some agro-industrial by-products can be valorized by their inclusion in feed formulations. Hazelnut skin is a by-product of the hazelnut industry. It is a good source of phenolic compounds, polysaccharides, unsaturated fatty acids, and vitamin E. In this study, we evaluated the productive performance of dairy cows in response to the inclusion of hazelnut skin in the diet. We observed no effects of hazelnut skin on either the milk production level or fat and protein contents. The oleic acid concentration in milk was strongly increased by including hazelnut skin in the cows’ diet. Improvements in the antioxidant activity and sustainability of milk production in terms of food–feed competition were also observed. These results are of practical application for feed manufacturers and farmers, as they support the inclusion of sustainable and low-cost feed ingredients in ruminant diets, with the aim of supporting the expected increase in livestock production in the upcoming years.

**Abstract:**

Hazelnut skin (HS) was evaluated as a source of nutrients for dairy cows. In total, 26 Aosta Red Pied cows were divided into two balanced groups. All cows were fed hay ad libitum. The control group was also given 6 kg of concentrate, while the hazelnut skin group (HAZ) was given 1 kg of the same concentrate that was substituted by 1 kg of HS. The dry matter intake of the cows was reduced by the dietary inclusion of HS (*p* ≤ 0.001). The milk yield and main constituents were unaffected by treatment. Milk from HAZ cows showed decreased concentrations of de novo saturated fatty acids (FAs), odd- and branched-chain FA, α-linolenic acid, and long-chain n-3 FAs, as well as increased concentrations of stearic acid, oleic acid, linoleic acid, total monounsaturated FAs, trans biohydrogenation intermediates, and α–tocopherol. Replacing the concentrate with HS increased the human-inedible feed quota in the diet and improved the sustainability of milk production in terms of the food-feed competition. Our results suggest that it is possible to add economic value to organic waste from the hazelnut industry using HS as a feed ingredient for dairy cows, enhancing the feed efficiency and milk antioxidant activity and having expected impacts on the nutraceutical quality of milk fat.

## 1. Introduction

Hazelnut (*Corylus avellana* L.; botanical family: Betulaceae) is the fifth most produced tree nut in the world, with an estimated rate of human consumption per capita of 0.06 kg year^−1^ [1]. In 2018, the global production of hazelnuts was slightly lower than 1 million tons [2]. Turkey is the world’s leading producer, with a harvested area equal to 728,381 ha that accounts for 58% of the world’s total production. Significant contributions to the world hazelnut trade are also made by Italy (15% of the total production and 11% of the total exports) and Azerbaijan (6% of the total production and 6% of the total exports) [2]. Hazelnuts are mainly consumed whole, with (raw) or without (roasted) their skin. In addition, hazelnuts are also used as an ingredient in many processed foods [3].

Different by-products originate from the hazelnut industry during the harvesting and post-harvesting processes. These include hazelnut tree leaves, hazelnut green leafy covers, hazelnut shells, and hazelnut skin (HS). These by-products are rich sources of phenolic compounds and have been shown to exhibit stronger antioxidant activities compared to the hazelnut kernel [4], and can also potentially be used as health-promoting ingredients [5]. The hazelnut industry is interested in the economic valorization of these by-products. However, currently only the hazelnut shells have direct commercial value as a heating source when burnt as a boiler fuel and as mulch [6]. Due to their biologically active compounds [7], hazelnut by-products have recently gained attention from many researchers worldwide who are attempting to find alternative uses for organic waste, and who are also trying to contribute to the desired transition towards a circular economy. For example, hazelnut shells have been suggested to be used as functional ingredients for food supplements, pharmaceutical and cosmetic formulations [8], and as substrates in biogas plants [9].

The HS (also named the “testa”, “peel”, or “hull”) is the perisperm of the hazelnut kernel, which represents approximately 2.5% of the kernel weight [10]. The skin is separated from the kernel in the last step of production (roasting) and is usually discarded, thus representing a problem from both environmental and economic perspectives [11]. The interest in HS is due to its bioactive substances [12]. Besides the high concentration of phenolic compounds [13,14], HS can also be considered a good source of polysaccharides [15,16,17] and total lipids, including unsaturated fatty acids (FA) (mainly oleic acid) and tocopherols [18]. Overall, HS has good energy density and among hazelnut by-products it has the strongest antioxidant properties [4,13].

Recent studies have evaluated the potential use of HS for the enhancement of the nutritional value of food for human consumption. These studies have shown that it is possible to use HS in the production of yogurt [19] and fresh egg pasta [17] in order to obtain fortified food with high fiber content and antioxidant activity. When used as an ingredient in the manufacture of chicken burgers, HS improved the cooking characteristics and antioxidant properties of the burgers, despite having a negative impact on the lipid stability during refrigerated storage [20]. In addition, microfluidized HS was shown to have potential use as an ingredient in bakery products [21]. As with many other agro-industrial by-products rich in polyphenols and tocopherols [22,23], HS may also be exploited as a feed source to enhance an animal’s serum oxidative status and immune function [24] and to produce animal-derived food products with functional nutritional value. Nevertheless, scant information is currently available on the topic. Cetinkaya and Kuleyin [25] evaluated the proximate composition, organic matter digestibility, metabolizable energy (through in vitro gas production), and antioxidant activity of the skins of different varieties of hazelnuts. Based on the obtained results, these authors suggested that HS may be considered as an alternative roughage source in ruminant nutrition or as a feed additive for both ruminants and monogastric animals. Only two very recent studies are currently available on the inclusion of HS in the diets of ruminants and on the related effects on animal product quality. Caccamo et al. [26] evaluated how the inclusion of HS in the diets of dairy ewes can affect the chemical and sensory characteristics of ovine cheeses, showing the by-product to have significant impacts on the lipid content and FA and sensory profiles of the cheeses. The replacement of dried beet pulp by HS in a commercial formulation for lactating ewes had no effects on feed intake and milk yield [27]. However, the milk protein percentage and somatic cells were reduced and the atherogenic index and health-promoting unsaturated FAs in milk were improved.

In the current study, we aimed at providing novel information on the use of HS as a feed ingredient for dairy cows by evaluating the related effects on feed intake, milk yield, milk main constituents, FA profile of milk fat, and the tocopherols content of the milk. Furthermore, we investigated the amount of potentially human-edible feeds in the experimental diets and assessed their net contribution to the human food supply.

## 2. Materials and Methods

### 2.1. Animals, Experimental Design, and Dietary Treatments

The experimental protocol was designed according to the guidelines of the current European Directive (2010/63/EU) on the protection of animals used for scientific purposes. All procedures and treatments were in compliance with the European Directive (Council Directive 98/58/EC) on the minimum standards for the protection of animals bred or kept for farming purposes.

The experiment lasted 48 days and was carried out at the Experimental Farm of the Institut Agricole Régional of Aosta, in Northwestern Italy (latitude: 45°43′59″ N, longitude: 7°18′2″ E; altitude, 560 m a.s.l.). In total, 26 mid-lactation multiparous Aosta Red Pied cows were selected from a herd of 60 lactating cows. The cows were divided into two balanced groups of 13 animals each, according to their stage of lactation (mean and standard deviation: 95 ± 28.9 days in milk), milk yield (19 ± 3.5 kg/head per day), milk main constituents (fat, protein, casein, and lactose contents), and FA profile of milk fat. The groups were then randomly assigned to a control or an experimental diet. The control group (CTRL) was fed 15 kg of mixed hay (first cut: 10 kg; second cut: 5 kg) and 6 kg of concentrate per head per day. The second group (hazelnut skin group, HAZ) was offered with the same type and amount of hays as CTRL, 5 kg of the same concentrate as CTRL, and 1 kg of HS. The concentrate contained (g/kg) wheat bran (280), barley (230), soybean 44% crude protein (CP) (120), beet pulp (120), soybean hulls (100), wheat (80), cane molasses (37), calcium bicarbonate (14), sodium bicarbonate (10), sodium chloride (5), and a min-vit premix (4). The by-product was mixed with water (1:1 *w*/*w*) and then with the concentrate, and was administered in equal amounts during the morning (6.00 h) and afternoon (18.00 h) milking’s.

All selected cows were housed indoors in individual pens and had free access to water.

### 2.2. Feed Intake, Sampling and Analysis

After 2 weeks of diet adaptation, feed refusals were weighed daily to determine individual feed intake. Representative samples of each feedstuff used in the trial were collected at the beginning and middle of the trial. They were ground with a cutting mill to pass a 1-mm screen sieve (Pulverisette 15-Fritsch GmbH, Idar-Oberstein, Germany). AOAC International [28] procedures were used to determine dry matter (DM, method no. 930.15), ash (method no. 942.05), CP (method no. 984.13), acid detergent fiber, and acid detergent lignin (ADF and ADL, method no. 973.18). Ether extract (EE, method no. 2003.05) was analyzed according to AOAC International [29]. Neutral detergent fiber (NDF) was analyzed according to Van Soest et al. [30]; α-amylase (Sigma Aldrich, Saint Louis, MO, USA), but no sodium sulphite, was added and results were corrected for residual ash content. Rumen-degradable protein (RDP) was analyzed according to Licitra et al. [31]. The energetic value of feeds was expressed as net energy for lactation (NE_L_) and was estimated using National Research Council (NRC) equations [32].

The FA composition of each feedstuff was assessed as described by Dabbou et al. [33]. Fatty acid methyl esters (FAME) were separated, identified, and quantified on the basis of the chromatographic conditions reported by Renna et al. [34]. The results were expressed as mg/100 g DM. The daily intake (g/head) of each individual FA and groups of FA from the diet was estimated considering the daily intake and the analytically determined FA composition of each feedstuff.

Standard protocols were used to assess the contents of total extractable phenols (TEP) and different polyphenol fractions (non-tannin phenols, NTP; condensed tannins, CT) in HS, as detailed in Iussig et al. [35]. Total tannins (TT) were computed as the difference between TEP and NTP. Hydrolysable tannins (HT) were estimated as the difference between TT and CT [36]. The amount of phenolic compounds daily ingested by the cows belonging to the HAZ group was estimated based on the analyzed phenolic composition of HS and the determined intake of concentrate.

Determining α- and δ-tocopherol in feeds was detailed in Cairoli [37] and adapted from Plozza et al. [38] and Lanina et al. [39]. Briefly, for saponification and extraction pyrogallol (7% *w*/*v* in ethanol), NaCl (1% *w*/*v* in distilled water), KOH (60% *w*/*v* in distilled water), and a mixture heptane: ethyl-acetate 9:1 (*v*/*v*) were used. The analyses were performed with a Varian 212-LC system (Varian Inc., Walnut Creek, CA, US) connected to an in-built Mass Spectrometer detector. The tocopherols were separated on a C18 Gemini-NX column (150 cm × 2 mm; 3 μm particle size) from Phenomenex (Lane Cove, New South Wales, Australia). The mobile phase consisted of acetonitrile (A) and 0.05% formic acid (B). Gradient elution of solvent A and B was: 10:90 *v*/*v* (0–15 min), 100:0 *v*/*v* (15–30 min), 10:90 *v*/*v* (30–42 min) at a flow rate of 200 μL/min and Vitamin K_3_ was chosen as the internal standard for quantifying the two tocopherols isomers.

All analyses were performed in duplicate.

### 2.3. Milk Sampling and Analysis

Individual milk yield was recorded and individual composite samples (n = 104) of morning and afternoon milking’s (proportional to milk production recorded per milking) were collected after 15, 26, 37, and 48 days from the beginning of the trial. Each milk sample was divided into three aliquots, immediately stored at 4 °C in a portable refrigerator, and transported to the laboratory. The first aliquot (50 mL) was analyzed for fat, protein, casein, lactose, and non-fat solids (MilkoScan FT 6000, Foss Electric, Hillerød, Denmark). The second aliquot (150 mL) was frozen at −80 °C and successively analyzed for the FA composition. Lipid extraction, FAME separation, identification, and quantification were performed as detailed in Cornale et al. [40]. The third aliquot (50 mL) was immediately stored in a dark bottle and frozen at −80 °C prior to α-tocopherol analysis. The analytical procedure for the determination of α-tocopherol in milk was the same described for the feed, with some modifications: (i) an isocratic elution was carried out with acetonitrile; (ii) the quantification of α-tocopherol was performed with δ-tocopherol as the internal standard.

All analyses were performed in duplicate.

### 2.4. Calculation of Feed Efficiency Parameters

Energy-corrected milk (ECM) was calculated for each cow using the following formula [41]:

ECM (kg) = [(kg of milk) × 0.327] + [(kg of fat) × 12.95] + [(kg of protein) × 7.2].(1)

Feed efficiency (FE) is defined as a unit of milk produced per unit of DM consumed and was calculated as ratio of ECM (kg) and dry matter intake (DMI, kg).

The human-edible feed conversion efficiency (heFCE) was defined as the ratio between human-edible output in the form of animal products (i.e., the milk in this study) and the potential human-edible input via feedstuffs [42]. Similarly, net food production (NFP) was calculated as the difference between the human-edible content in the milk and the potential human-edible content in the feed consumed. The two indexes were expressed on a CP and gross energy (GE) basis, and they were calculated using the estimated human-edible proportions proposed by Wilkinson [43] for the feedstuffs. Data on GE content of feedstuffs were derived from the INRA database [44]. Data on GE amount of milk was calculated according to NRC (32) using the following formula:

Milk-energy (MJ/d) = ([0.384 × fat (%)] + [0.223 × protein (%)] + [0.199 × lactose (%)]) × milk yield (kg/d).(2)

### 2.5. Statistical Analysis

Data were statistically analyzed using SAS software, version 9.4 (SAS Institute Inc., Cary, NC, USA). The changes in DMI, fatty acids, tocopherols intake, milk yield, and milk composition were analyzed using the MIXED procedure for repeated measures over time. The cow was considered as the experimental unit. Compound symmetry, first order autoregressive, or unstructured covariance structure, according to the smallest Schwarz Bayesian information criterion, was applied [45]. The following model was used:

Y_ijk_ = μ + DT_i_ + C_(i)j_ + SD_k_ + (DT × SD)_ik_ + ε_ijk_ (3)

where Y_ijk_ = mean of response variable, μ = population mean, DT_i_ = fixed effect of dietary treatment, C_(i)j_ = random effect of cow within the treatments, SD_k_ = fixed effect of sampling date, (DT × SD)_ik_ = fixed effect of interaction between dietary treatment and sampling date, and ε_ijk_ = experimental error. Significance was set at *p* ≤ 0.05. Results are reported as estimate least-squares means.

## 3. Results

### 3.1. Chemical Composition of Hazelnut Skin and Experimental Diets

The proximate and FA compositions, as well as the tocopherols concentration of the feedstuffs and of the experimental diets, are reported in Table 1.

The hazelnut skin used in this trial showed high ether extract (224 g/kg DM) and fiber (NDF: 530 g/kg DM; ADF: 492 g/kg DM) concentrations, while the CP and lignin concentrations were low (62 and 24 g/kg DM, respectively). The NE_L_ of the by-product was equal to 2.58 Mcal/kg DM.

The experimental diets were almost isonitrogenous (121 and 115 g/kg DM for the CTRL and HAZ diets, respectively). The HAZ diet showed slightly higher ether extract concentration (37 vs. 27 g/kg DM) and NE_L_ (1.33 vs. 1.28 Mcal/kg DM) when compared to the CTRL diet.

As far as the FA composition is concerned, HS showed a prevalence of monounsaturated fatty acids (MUFA; 175.92 g/kg DM, corresponding to the 80.44% of total detected FA (TFA)). Oleic acid (C18:1 *c*9) was by far the most abundant individual FA in HS (97.48% of total MUFA and 78.41% of TFA). Almost comparable amounts of saturated fatty acids (SFA) and polyunsaturated fatty acids (PUFA) were detected in the by-product (18.49 and 24.30 g/kg DM, respectively), comprising the 8.45% and 11.11% of TFA, respectively. The second and third most abundant individual FA in HS were linoleic acid (C18:2 *c*9*c*12) and palmitic acid (C16:0), which accounted for 10.95% of TFA (98.56% of total PUFA) and 6.24% of TFA (73.77% of total SFA), respectively. Alpha-linolenic acid (C18:3 *c*9*c*12*c*15) was only detected in very low amounts of HS (<0.5% of TFA).

The experimental diets were quite different in terms of their FA composition. The main dissimilarity regarded the concentration of oleic acid (3.49 and 11.89 g/kg DM for the CTRL and HAZ diets, respectively). The relative abundance of the main groups of FA was also quite different. The CTRL diet mainly contained PUFA (48.74% of TFA), followed by SFA (27.73%), and finally by MUFA (23.54%). On the contrary, in the HAZ diet, MUFA represented almost the half (47.44%) of TFA, followed by PUFA (32.93%), and finally by SFA (19.60%).

The recorded amount of TEP in HS was remarkable (265 g/kg DM) (data not reported in Table 1). More than the half of TEP was represented by tannins (TT equal to 158.51 g/kg DM), the majority of which were hydrolysable (HT and CT equal to 89% and 11% of TT, respectively). Polyphenols were not determined in hays and concentrate, as their amounts were expected to be negligible when compared to the amounts found in HS [27].

As expected, HS showed noticeably higher levels of α- and δ-tocopherols compared to the other feed ingredients. The concentration of α–tocopherol in HS (153.74 mg/kg DM) was 4.3-fold higher than in the concentrate and about 6-fold higher than in the hays (35.68, 25.26, and 23.93 mg/kg DM, respectively). The differences in δ-tocopherol amounts among the feeds were even more remarkable, ranging between 1.17 mg/kg DM in the concentrate and 67.28 mg/kg DM in the HS. The inclusion of HS thus increased α–tocopherol (+22.41%) and δ-tocopherol (+112.83%) concentrations in the HAZ diet when compared to the CTRL diet.

### 3.2. Intake of Dry matter, Fatty Acids, Phenolic Compounds, and Tocopherols

The effects of the dietary inclusion of HS on DM, FA, and tocopherols intake by the cows are presented in Table 2.

The dietary treatment had no significant effect on the individual intake of concentrate, which was always totally ingested by the cows in both experimental groups. However, a significant decrease of the individual intake of hay was observed with the inclusion of HS in the diet (−11.43%; *p* < 0.001). Consequently, the individual total DMI of the cows was significantly reduced with the dietary inclusion of HS (−7.38%; *p* < 0.001).

The statistical analysis showed highly significant differences (*p* < 0.001) between the two groups of cows for the intake of all individual FA and groups of FA from the diets. In particular, the HAZ group of cows showed a noticeably higher intake of oleic acid (+153.50 g/head × day) and slightly higher intakes of C16:0 (+3.96 g/head × day), stearic acid (C18:0; +2.86 g/head × day), and linoleic acid (+10.68 g/head × day), as well as contemporarily lower intakes of α-linolenic acid (−5.97 g/head × day) when compared to the CTRL group.

Similarly, the daily intake of tocopherols was different between the two experimental groups. The cows fed the HAZ diet showed higher (*p* < 0.001) intakes of both α- (+84.15 mg/head × day) and δ-tocopherol (+59.63 mg/head × day), increasing the daily intake by 17.67 and 119.16% when compared to the CTRL group, respectively.

The amounts of TEP, NTP, TT, CT, and HT daily ingested by the cows belonging to the HAZ group were equal to 253.17, 101.79, 151.38, 16.59, and 134.79 g/head × day, respectively (data not reported in Table 2).

Sampling date significantly affected hays intake, total DMI, and the intake of all individual FAs and tocopherols. For the majority of parameters, the lowest values were observed at the second sampling date.

The DT × SD interaction did not significantly affect DM, FA, or tocopherol intake.

### 3.3. Milk Yield and Milk Main Constituents

The effects of the dietary inclusion of HS on milk yield, energy corrected milk, milk fat, protein, casein, lactose contents, and yields are presented in Table 3.

Individual milk yield and energy corrected milk were not affected by treatment. A lack of significant effects of dietary inclusion of HS was also observed for the milk main constituents, in terms of both contents and yields. The protein content in milk tended (*p* = 0.08) to be lower for HAZ cows, but the extent of the difference (−2.47%) was small.

Milk fat and protein percentages increased over time in both experimental groups (data not shown).

The DT × SD interaction was not significant for the considered parameters.

### 3.4. Milk Fatty Acid Profile

The inclusion of HS in the diet significantly affected the FA composition of cow milk fat (Table 4, Table 5 and Table 6).

The concentration of total SFA and those of almost all individual SFA were significantly lower in the milk from the HAZ group when compared to the milk from the CTRL group (Table 4). Exceptions regarded butyric acid (C4:0), C18:0 and eicosanoic acid (C20:0). The latter was unaffected by the treatment, while both C4:0 and C18:0 showed a significantly higher concentration in the HAZ than CTRL group (*p* < 0.05 and *p* < 0.001, respectively).

The milk concentrations of all individual odd-chain fatty acids (OCFA) and branched-chain fatty acids (BCFA) were also negatively affected by the inclusion of HS in the cow diet (Table 4). Reductions ranged from 9.38% (C14 *iso*) to 50.00% (C18 *iso*). Total *iso* and total *anteiso* BCFA were reduced by 14.47% and 17.41%, respectively (*p* ≤ 0.001).

Different from what was observed for the total SFA, the concentration of the total MUFA significantly increased (+52.35%; *p* < 0.001) in HS-fed cow milk when compared to the milk of the cows fed the CTRL diet (Table 5). Significant increases were observed for the concentration of oleic acid (+56.00%; *p* < 0.001) and for the concentrations of all individual octadecenoic isomers (both the *cis* and *trans* forms). Consequently, the concentrations of total octadecenoic and total *trans*-octadecenoic acids were noticeably enhanced with the inclusion of HS in the cow diet (+59.01% and +87.20%, respectively; *p* < 0.001). Some individual MUFA (i.e., C14:1 *t*, C20:1 *t*, and C20:1 *c*11) were not affected by the dietary treatment, while the concentration of MUFA with a *cis*-9 double bond and a number of carbon atoms up to 16 significantly decreased with the inclusion of HS in the diet.

The milk concentration of total PUFA slightly increased with the HS inclusion in the diet (+8.04%; *p* < 0.001; Table 6). Linoleic acid and octadecadienoic acids mainly deriving from its biohydrogenation were significantly increased (C18:2 *c*9*t*12, C18:2 *c*9*t*11 + *t*7*c*9 + *t*8*c*10) or not affected (C18:2 *t*9*c*12, C18:2 *t*10*c*12, C18:2 *t*9*t*11) by the inclusion of HS in the cow diet. On the contrary, α-linolenic acid and octadecadienoic acids mainly deriving from its biohydrogenation were significantly decreased (C18:2 *t*11*c*15, C18:2 *c*9*c*15) or not affected (C18:2 *t*11*c*13 + *c*9*c*11) by the dietary inclusion of HS. Overall, the concentrations of the total octadecadienoic acids, the total *trans*-octadecadienoic acids, and the total conjugated linoleic isomers (CLA) were significantly higher in the milk of the cows fed HS when compared to the cows fed the CTRL diet (+18.49%, +22.04% and +24.71%, respectively; *p* < 0.001).

Total omega-3 PUFA and individual detected long-chain omega-3 PUFA (EPA and DPA) were reduced in the milk of the HS-fed cows. DHA was not detected in the analyzed milk samples. The omega-6 to omega-3 PUFA ratio of the milk worsened with the dietary inclusion of HS (2.34 vs. 3.61 for the CTRL and HAZ groups, respectively).

The sampling date significantly tended to affect many of the detected FA. The majority of these changes were small and occurred during the experimental period (weeks 15–20 into lactation) without any clear increasing or decreasing trend (data not shown).

The DT × SD interaction was not significant for the considered parameters.

### 3.5. Milk Tocopherol Content

The higher tocopherols content in the HAZ diet and the resulting higher intake of tocopherols by the cow fed HS were reflected in the milk. Hence, the HS inclusion in the diet significantly affected the α-tocopherol concentration in milk. The cows fed HS showed higher (+21.38%; *p* < 0.05) α–tocopherol concentration in milk when compared to the CTRL group (20.67 and 25.09 mg/kg fat, respectively).

The tocopherol content in milk increased over time in both experimental groups (data not shown) and was not significantly affected by the DT × SD interaction term.

### 3.6. Feed Conversion Efficiency and Diet Cost

The results about feed efficiency parameters, net food production, and diets cost are showed in Table 7.

The cows belonging to the HAZ group showed a significantly higher feed efficiency when compared to the cows belonging to the CTRL group (+11.21%; *p* < 0.01).

The inclusion of HS in the diet affected heFCE in terms of both CP and GE (*p* < 0.001), with values above and below 1 for the HAZ and CTRL diets, respectively. Similarly, NFP in terms of both CP and GE showed positive values for the HAZ diet, while the index showed negative values for the CTRL group (*p* < 0.001). These results showed that the cows fed the CTRL diet consumed more human-edible protein and energy than they produced in the milk.

The cost of the diets consumed by the cows during the trial were calculated using the market price of feeds. The inclusion of HS, coupled with the reduction of hay intake, significantly (*p* < 0.001) reduced the daily cost of the HAZ diet compared to the CTRL diet by 8.36% (3.47 vs. 3.18 €/cow, respectively).

Since feed efficiency and diets cost are calculated based on DMI, they were also significantly affected by sampling date. The lowest values were observed at the second sampling date. The DT × SD interaction was not significant for the considered parameters.

## 4. Discussion

### 4.1. Chemical Composition of Hazelnut Skin

The HS used in this trial showed a proximate composition very similar to that described in the available published literature [17,26]. In accordance with the results of these authors, the major component in HS was fiber (>500 g/kg DM). The EE content was also high, falling within the ranges (109.9 to 287.8 g/kg DM) reported by Zeppa et al. [17] for four different cultivars. The CP content of HS was low, being comparable to values reported by Cetinkaya and Kuleyin [25] (around 60 g/kg DM) and slightly lower than the values found by Caccamo et al. [26] and Longato et al. [20] (74 to 79 g/kg DM). Campione et al. [27] also reported that around the 68% of protein in HS is in the form of unavailable nitrogen (acid detergent insoluble protein).

As far as the FA composition of HS is concerned, few data are currently available in the literature, and published profiles are very simple in terms of individual identified FA. We provided a more detailed FA profile of HS, showing the presence of *cis*-vaccenic acid (C18:1 *c*11) and behenic acid (C22:0), which were not previously identified in this by-product. Overall, the obtained FA profile confirms the recent findings of Ivanović et al. [46] and Campione et al. [27], with HS showing a high content of MUFA, with a clear prevalence of oleic acid, followed by linoleic and palmitic acids. These three FA comprised the 96% of TFA, as also obtained by the above-mentioned authors.

Hazelnut is a rich source of phenolic compounds and many published papers investigated the total phenolic content and the phenolic composition of this edible nut and its by-products ([7], among many others). Del Rio et al. [14] found an average content of total polyphenols equal to about 675 mg/100 g after analyzing with HPLC-MS/MS and the skin of nine different multicultivar and monocultivar samples from Turkey, Italy, and Chile were found. Zeppa et al. [17] reported a total average phenolic content of the skin of four different varieties of hazelnut ranging from 102.19 to 195.76 mg of gallic acid equivalents per gram of dry weight. Because of the use of different extraction methods and/or different expressions of the obtained results, it is difficult to compare our results with those previously obtained by other authors. However, with comparable analytical methods used in our trial, Campione et al. [27] found amounts of TEP and TT in HS equal to 132 and 76.7 g/kg DM. Such values were about half of the values we found in the current trial. Such discrepancies could be due the hazelnut varieties employed in the different trials. In fact, concentrations of phenolic compounds are clearly distinct and vary consistently (up to almost 4 times) among hazelnut varieties [13].

Nuts contain high amounts of fat-soluble bioactives with antioxidant activity and they are mainly located in the perisperm [47]. Among all nuts, hazelnuts have been reported to have the highest tocopherols content [48], with skin containing almost double amount of tocopherols than kernel [13]. While several studies investigated tocopherol content in hazelnuts, limited data about the tocopherols content in hazelnut skins are available. Taş and Gökmen [13] assessed the tocopherols content in the skin of 14 Turkish hazelnut varieties. The α-tocopherol was detected as the dominant fraction and the content was found to range from 168.2 to 443.8 mg/g. The HS used in our study showed comparable (although slightly lower) content of α-tocopherol. Besides differences in analytical procedures, hazelnut varieties and harvest time have been reported as significant factors influencing the tocopherols content [49]. Regarding the effect of roasting on tocopherols content in hazelnut and its skin, contrasting results are available in literature [50].

### 4.2. Dry Matter Intake, Milk Yield, and Milk Main Constituents

The inclusion of HS in the diet depressed the total DMI of the cows (Table 2). Tannins may reduce feed palatability due to the binding to salivary glycoproteins, determining an unpleasant feeling of dryness and harshness [51]. In our trial, the concentrate was always fully eaten by the cows (Table 2). Such result demonstrates the lack of negative effects of low inclusion levels of HS (about 6% of DMI) on feed palatability in dairy cows. Considering that the HS used in the present trial mainly contained HT, our results are in agreement with the findings of Costa et al. [52] who showed higher diet palatability when hydrolysable tannin extract from chestnut instead of condensed tannin extract from mimosa was incorporated into oil-supplemented diets fed to sheep. However, tannins also directly inhibit cellulolytic microorganisms and fibrolytic enzymatic activity and increase rumen physical filling, because of the formation of complexes with lignocellulose that depress fiber digestion [53]. These phenomena could be the reason of the observed reduction of hay intake and, consequently, of total DMI, in our trial. Anyway, it is worth noting that tannin inclusion in the diet of ruminants has shown contrasting results in terms of DMI in published literature. Such discrepancies can be attributed to variations in basal diet, concentration in diet, tannin source (and thus chemical structure and molecular weight), and other animal-related factors [54].

Overall, in our trial, no significant effects were observed on milk yield and milk fat, protein, casein, lactose percentages, and yields, while including HS in the cow’s diet (F:C ratio of the diet equal to 66:34). Similar results were also obtained by Campione et al. [27], who compared the production performance of Comisana lactating ewes fed a diet (F:C equal to 69:31) including alfalfa hay ad libitum plus 800 g/head/day of a pelleted concentrate containing 36% dried beet pulp or HS.

In ruminants, an increase in the fat content of the diet within certain limits leads to increased percentages of fat in milk and derived cheese [55]. Consistently, Campione et al. [27] found a tendency towards a higher percentage of fat in the milk from HS-fed ewes. In that trial, the concentrate containing HS had 5.6 times higher EE than the control concentrate. When analyzing Pecorino cheese derived from the same ovine milk, Caccamo et al. [26] reported a significantly higher lipid content in the cheese manufactured using the HS-derived milk. In our trial, the inclusion level of HS in the cow diet was about half of that used by Campione et al. [27] (6.0% vs. 11.3%) and increased the EE content of the whole diet by 37%. This led to a slight increase of the milk fat content (+2.80%) and yield (+5.13%) when the cows were fed HS, but the difference between CTRL and HAZ groups was not statistically significant.

Despite comparable CP contents of the experimental diets, Campione et al. [27] found a negative effect of dietary HS on the protein percentage of ewe’s milk. At the same time, in that trial, no variations were observed regarding the percentage of individual milk proteins, with the exception of α-lactalbumin that was negatively affected by the inclusion of HS in the diet. Our data, with a tendency towards a reduction of the protein content of milk obtained from the HS-fed cows, seem to corroborate the findings of Campione et al. [27], even if the extent of variation was small. These authors hypothesized that the lower milk protein percentage found in the HS-derived milk could be the consequence of a higher acid detergent insoluble protein, higher acid detergent lignin, and lower non-fiber carbohydrates content found in the HS and not the control concentrate, leading to an unfavorable ratio between soluble protein and non-fiber carbohydrates in the HS diet. Due to its high acid detergent insoluble protein content linked to lignin and due to Maillard reaction products originated during roasting [56], HS is characterized by low rumen degradability. In fact, acid detergent insoluble protein is undegradable in the rumen and not digested by proteolytic enzymes in the small intestine, being therefore unavailable for protein synthesis [57]. The chemical analysis conducted on the HS used in our trial confirmed that the CP in HS (62 g/kg DM) is mostly undegradable inside the rumen (RDP = 5.0 g/kg DM CP). Another aspect to be considered is the presence of plant secondary metabolites in the by-product. Evaluating the effects of CT from HS on in vitro rumen fermentation parameters, recently Niderkorn et al. [58] showed that HS resulted in a lower protein degradation and, consequently, in a decreased NH_3_ concentration in the fermentation medium. Forming complexes with proteins, CT protect proteins from ruminal degradation. The protein binding activity of CT depends on their prodelphinidin/procyanidin ratio. Niderkorn et al. [58] demonstrated that CT in HS are characterized by a low prodelphinidin/procyanidin ratio, which makes them less active in protein binding activity when compared to CT from other feeds. The low dietary inclusion level of HS in our trial affected the overall protein degradability of the diet only slightly (RDP = 83.7 vs. 77.4 g/kg DM in the CTRL and HAZ diets, respectively). This could be the reason of the less intense negative effect of HS on cow milk protein percentage in our trial when compared to what observed by Campione et al. [27] in dairy ewes (protein percentage reduced by 2.47% and 6.76%, respectively). It should also be pointed out that, in our trial, the adopted inclusion level of HS led to a ratio between rumen degradable and rumen undegradable (RUP) protein of about 68:32. Such an RDP:RUP ratio can be considered as well balanced for optimum rumen function and fulfilment of nitrogen needs in the Aosta Red Pied, which is a dual-purpose medium producing cow breed [32,59].

### 4.3. Milk Fatty Acid Profile

As far as milk fatty acid composition is concerned, the most evident result of the inclusion of HS in the cow diet was the noticeable increase of MUFA (Table 5). Such result was expected, being that HS is very rich in oleic acid (Table 1). Even low inclusion levels of HS in the diet, as occurred in our trial, intensely increased the intake of both oleic acid and total MUFA by the cows (Table 2). This led to an increase in milk fat of not only oleic acid but also of all other *cis*- and *trans*-octadecenoic acids (Table 5), which may partly derive from oleic acid isomerization operated by the microflora inside the rumen [60]. Another explanation for the increase of *trans*-octadecenoic isomers in milk is the presence of tannins in HS. Several studies showed that tannins are able to modulate rumen biohydrogenation, leading to increased concentrations of biohydrogenation intermediates, particularly C18:1 *trans*-isomers [61]. Costa et al. [52] suggested that HT cause a higher accumulation of *trans*-octadecenoic acids in the rumen when compared to CT. It is also known that tannins can two-fold increase the concentration of vaccenic acid in digesta. The same increase is instead usually lower than 100% in milk, as vaccenic acid can escape complete ruminal biohydrogenation, then being desaturated to rumenic acid within the mammary gland [54]. Consistently, we observed a significantly higher concentration of rumenic acid (which coeluted with C18:2 *t*7*c*9 and C18:2 *t*10*c*12) in the milk from the cows fed the HAZ- rather than the CTRL-diet (Table 6).

Besides the increases of total MUFA, oleic acid, *trans*-octadecenoic isomers, and CLA, another clear effect of the dietary inclusion of HS was the decrease of odd- and branched-chain fatty acids (OBCFA) in milk. Such a result is in complete agreement with the recent findings of Campione et al. [27], who observed significant reductions of all individual OBCFA in rumen content and milk fat from lactating ewes fed a diet containing HS. OBCFA are major lipids of bacterial membranes used as important diagnostic parameter for rumen microbial activity. The observed decrease of OBCFA in milk from HS-fed cows can be attributed to the presence of tannins in HS. Great variability is reported in the literature about the response of FA from its microbial origin to dietary tannins [54]. Buccioni et al. [62] showed that the amount of BCFA significantly decreased in milk fat from ewes fed either CT from quebracho and HT from chestnut. OBCFA response to dietary tannins is dose-dependent. Anyway, considerable variations in the concentrations of OBCFA in digesta and milk were also reported when tannins were included in low amounts (≤3% in diet), suggesting important variations in rumen bacterial populations [54].

Tannins have also been commonly reported to decrease the biohydrogenation extent of common (e.g., C18:2 *c*9*c*12 and C18:2 *c*9*c*12*c*15) and less common (e.g., C18:3 *c*9*t*11*c*13 and C18:3 *c*9*t*11*t*13) PUFA in vegetable feeds, leading to desirable increased concentrations of these FA in rumen digesta and milk fat [54]. In our trial, while linoleic acid concentration increased in milk fat from HS-fed cows, the opposite was observed for α-linolenic acid. Moreover, some intermediates of ruminal biohydrogenation followed the same trend of their precursors (Table 6). The obtained results seem most probably the effect of the higher and lower intake of C18:2 *c*9*c*12 and C18:3 *c*9*c*12*c*15 in the cows fed the HAZ diet than CTRL diet, respectively (Table 2), and not a direct consequence of the presence of tannins in the by-product.

Overall, the effect of the inclusion of HS in the diet of dairy cows on the FA profile of bovine milk fat was very similar to what was recently observed in ovine milk FA from HS-fed dairy ewes [26,27].

The observed increase of oleic acid in cow milk fat is a positive outcome of the inclusion of HS in the animals’ diet. Oleic acid is known to possess beneficial effects on human health, especially on cancer, autoimmune diseases, inflammatory diseases, and wound healing [63]. In addition, the observed significant increase of total *trans*-octadecenoic isomers in the milk from HS-fed cows should not be of concern. Both in vitro and in vivo studies showed that *trans*-FA from ruminants do not increase the risk of developing cardiovascular diseases in humans, as it is instead observed for industrial *trans*-FA [64]. Such differences are due to a dissimilar isomeric distribution of the two fat sources. In milk and dairy products from ruminants fed high forage diets (as occurred in our trial), vaccenic acid is the most abundant among *trans* monoenoic acids. In humans, vaccenic acid is associated with a reduction of the risk of metabolic syndrome [64]. Similar beneficial effects, with particular reference to type 2 diabetes, have also been demonstrated for C16:1 *t*9 [65], which significantly increased with the inclusion of HS in the cow diet. With the chromatographic conditions applied in our trial, *t*10 and *t*11 C18:1 isomers coeluted. Their sum represented the 57.97% and 49.52% of total *trans*-octadecenoic isomers in HS and CTRL milk, respectively, suggesting a shift of the double bond position of *trans*-octadecenoic isomers in milk while adding HS to the ruminant diet. When analyzing the results obtained by Campione et al. [27], a similar shift also occurred in milk from HS-fed ewes and merits further consideration in future trials. The increase in milk concentration of rumenic acid is another positive effect of the dietary inclusion of HS on the FA composition observed in the present trial. Pre-clinical and human studies conducted using CLA to date collectively suggest that CLA has efficacy against cancer, obesity, and atherosclerosis [66].

Negative effects on the FA profile of milk fat (i.e., the reduction of OBCFA concentrations and the increase of the n6/n3 FA ratio) were also observed while including HS in the cows’ diet. OBCFA are important bioactive components exerting essential roles in the gut [67] and potential activity against human breast cancer cells [68]. The n6/n3 FA ratio significantly increased while including HS in the cows’ diet, as also occurred in milk from dairy ewes fed HS [27]. However, in both trials, this ratio remained within recommended values for human consumption [69]. It cannot be excluded that higher dietary inclusion levels of HS may determine further negative increases of this ratio in ruminant milk fat.

### 4.4. Milk Tocopherol Content

Milk contains a remarkable array of bioactive substances. These “minor” components, along with main constituents (lipids and proteins), contribute to the quality of milk and dairy products [70]. Vitamin E (and other fat-soluble vitamins) represents an important bioactive micronutrient of the lipid fraction in milk. The eight isomers of vitamin E differ in biological activity and α-tocopherol is the main form present in cow milk, representing approximately 90% of the total tocopherols [71]. Vitamin E is a potent antioxidant and a free radical scavenger. It inhibits the oxidation of polyunsaturated fatty acids preventing fat globules membrane degradation and consequently off-flavor development (e.g., rancidity) in milk. This is particularly helpful for milk that has been increased in its polyunsaturated lipid content aiming at making it healthier [72]. Therefore, vitamin E supplementation to ruminants has two main beneficial effects: it can support animal health that improves immune responses [73] and it increases the vitamin content in milk [74].

The α-tocopherol content in bovine milk has been reported to vary between 0.2 and 1.0 mg/L [75]. Several factors affect vitamin E content in milk such as breed, stage of lactation, production level, health status, season, and dietary regimen [76]. The levels of α-tocopherol obtained in the present study are consistent with the published literature, with average values equal to 0.73 and 0.90 mg/L in the CTRL and HAZ group, respectively.

Different feeding strategies affect the vitamin E content in cows’ milk [77]. Marino et al. [78] reported α-tocopherol contents in milk from grazing cows equal to 0.5–0.7 mg/L. Cows grazing at pasture produced milk richer in vitamin E than cows housed indoors and fed mainly with conserved forage (approximately 0.8 and 0.4 mg/L, respectively) [79]. However, Slots et al. [80] suggested that differences in tocopherols levels in milk obtained from pasture-fed cows could be due to the lower daily milk yield. Cows fed grass-clover silage showed higher level of α-tocopherol in milk than cows fed corn silage (0.85 vs. 0.38 mg/L, respectively) [81]. In a following study [72], the same authors did not find significant differences between cows fed grass-white clover silage and meadow hay (0.47 and 0.50 mg/L, respectively).

Numerous studies investigated the recycling of vegetable by-products as feed for ruminants [82]. Several of these by-products (e.g., from tomato, olive, and grape) are well known to be rich sources of tocopherols [23,83]. However, most of these studies did not assess the vitamin E content in the by-products and their effects on milk. The authors of the two studies examining the use of HS as feed in dairy ewes [26,27] did not report vitamin E analysis of feed and milk, pointing out the importance of such assessment in future studies.

There is an increasing attention about the use of by-products in animal feeding [82] and how these functional feeds can improve products quality and ameliorate animal health [84]. Although milk and dairy products provide a small percentage of human daily intake of vitamin E [85], it has been demonstrated that milk is an excellent nutrient delivery medium because it is able to increase the bioavailability and absorption of vitamin E [86]. The results of the present study, coupled with the fact that they were obtained through a natural supplementation (i.e., using a by-product naturally rich in RRR-α-tocopherol form and fed to cows as raw ingredient in the diet), validate HS as an effective feed ingredient in the diet of dairy cows able to increase the α-tocopherol content of milk.

### 4.5. Feed Conversion Efficiency

In the dairy sector, a high feed efficiency refers to the ability of cows to produce high output (milk) using low input (feed). However, this index does not take into account the type of feed fed to cows, only its amount. Nowadays, large amounts of cereal- and legume-based concentrates are used worldwide in conventional dairy systems to meet high nutrients requirements of high producing dairy cows. However, cereal grains and legumes could instead be used more efficiently by monogastric animals or be consumed directly by humans. On the other hand, ruminants, thanks to the microbiota populating the rumen, are able to use fibrous feedstuffs that are unsuitable for human consumption, converting them into high valuable and nutrient-dense food (i.e., milk and meat) [87]. Hence, reducing the inclusion of human-edible crops and feedstuffs in animals’ diets leads to a decrease in food–feed competition, representing a promising way to increase the sustainability of livestock production [88].

Along with grass-based feeding, the use of by-products in an animal diet is an effective feeding strategy to make dairy systems more sustainable [43]. Ertl et al. [89] firstly determined heFCE and NFP of dairy cows fed a by-products-based concentrate (a mixture of corn middlings, beet pulp, rapeseed, and soy cakes). The reduction of human-edible inputs by substituting common concentrates with the by-products improved heFCE and NFP (from 1.5 to 5.3 times). The overall amount of the by-products included in the ration was equal to 22.7% of DMI. Similarly, Pang et al. [90] significantly improved the human-edible feed conversion ratio for both energy and protein in dairy cows fed a by-products mixture (containing sugar beet pulp, wheat bran, canola meal, and others), combined with a grass silage. The inclusion of the by-product’s mixture exceeded the 30% of DMI. In another trial, Karlsson et al. [91] compared a conventional diet and three experimental diets containing by-products-based concentrates (representing about 42% of DMI). These authors obtained higher heFCE and net food production for both energy and protein in the by-product-based diets than in the control diet. The inclusion of HS in the present study seems to have affected heFEC and NFP at a lesser extent than the above-mentioned studies. However, it is worth mentioning that our results have been obtained with the inclusion of HS at a small percentage (about 6%) of DMI, a considerably lower amount if compared to the above-mentioned studies.

More recently, Bonanno et al. [92] replaced cereal grains and legumes in the diet of dairy cows with durum wheat bran, at 6% and 13% of DMI. The by-product also affected heFCE and NFP, with the best results at the higher inclusion rate. However, NFP for both energy and protein showed negative values in the control and experimental diets.

Sustainability is a complex concept that includes several aspects. Food–feed competition is one of the themes of livestock sustainability and it has recently seen an increasing interest especially in ruminant nutrition. The feed conversion efficiency indicators (heFCE and NFP) assess the potential human-edible content in animal diets, thus providing an index of sustainability. Confirming previous findings, our results demonstrate that replacing cereal grain and legumes with by-products in the diet of dairy cows increases the sustainability of dairy systems without lowering milk production.

## 5. Conclusions

These first insights suggest that it is possible to add economic value to waste from the hazelnut industry using low levels of hazelnut skin to increase the energy density of diets destined to dairy cows, without any negative effect on milk yield and main constituents.

Expected significant impacts on the nutraceutical quality of the lipid fraction of milk fat are due to both the fatty acid and phenolic compositions of the by-product. Such impacts are partly positive, such as the increase of oleic, *trans*-vaccenic, and rumenic acids, or the decrease of medium-chain saturated fatty acids. At the same time, negative effects are observed, as the overall increase of *trans* fatty acids, the increase of the omega-6 to omega-3 fatty acids ratio, and the decrease of omega-3 fatty acids in the milk.

Hazelnut skin possesses a strong antioxidant asset and we demonstrated that the inclusion of this by-product in the diet of dairy cows can significantly enhance the α-tocopherol content of cow milk. Furthermore, the inclusion of hazelnut skin in the cow’s diet, although at a small percentage of DMI, significantly improves the sustainability of the obtained milk in terms of food–feed competition and reduces the daily cost of the diet.

Further research is needed to assess the optimum dietary inclusion levels of hazelnut skin as to enhance the antioxidant activity of milk without having any impairment of the production performance of the cows. Applied research is also needed to enable the commercial application of hazelnut skin to livestock nutrition.

## Figures and Tables

**Table 1:** Proximate composition, fatty acid profile, and tocopherols content of the experimental feedstuffs and diets.

	Feedstuffs				Diets ^1,2^	
Parameter	Hay 1st Cut	Hay 2nd Cut	Concentrate ^3^	HS	CTRL	HAZ
Main nutrients (g/kg DM) unless otherwise stated)						
DM (g/kg)	893	881	882	955	887	890
Ash	79	119	75	23	87	85
CP	77	127	192	62	121	115
RDP (% CP)	66.8	69.5	70.1	8.0	69.2	67.3
EE	23	27	34	224	27	37
NDF	603	528	275	530	492	504
ADF	379	354	127	492	301	319
ADL	41	44	23	240	37	37
NSC ^4^	217	199	425	160	273	259
NE_L_ (Mcal/kg DM)	1.15	1.29	1.48	2.58	1.28	1.33
Fatty acids (g/kg DM)						
C12:0	0.13	0.28	0.01	n.d.	0.13	0.13
C14:0	0.16	0.20	0.06	0.14	0.14	0.14
C16:0	2.50	3.34	5.19	13.64	3.46	3.91
C16:1 *t*3	0.17	0.28	n.d.	n.d.	0.15	0.15
C16:1 *c*9	0.01	0.01	0.03	0.07	0.02	0.02
C18:0	0.29	0.35	1.00	4.36	0.51	0.68
C18:1 *c*9	0.69	0.51	10.70	171.48	3.49	11.89
C18:1 *c*11	0.06	0.06	0.45	4.05	0.17	0.36
C18:2 *c*9*c*12	2.42	2.69	10.04	23.95	4.65	5.40
C18:3 *c*6*c*9*c*12	0.03	0.04	0.01	n.d.	0.03	0.03
C18:3 *c*9*c*12*c*15	3.43	5.70	0.86	0.35	3.24	3.20
C20:0	0.16	0.12	0.09	0.26	0.13	0.14
C20:1 *c*9	n.d.	n.d.	n.d.	0.32	0.00	0.02
C22:0	0.14	0.14	0.10	0.10	0.13	0.13
Σ SFA	3.38	4.42	6.45	18.49	4.50	5.13
Σ MUFA	0.92	0.86	11.18	175.92	3.82	12.42
Σ PUFA	5.88	8.43	10.90	24.30	7.91	8.62
TFA	10.18	13.71	28.54	218.71	16.23	26.18
α-tocopherol	23.93	25.26	35.68	153.74	27.58	33.76
δ-tocopherol	2.05	7.31	1.17	67.28	3.04	6.47

Abbreviations: HS, hazelnut skin; DM, dry matter; CP, crude protein; RDP, rumen degradable protein; EE, ether extract; NDF, neutral detergent fiber; ADF, acid detergent fiber; ADL, acid detergent lignin; NSC, non-structural carbohydrates; NE_L_, net energy for lactation; n.d., not detected; SFA, saturated fatty acids; MUFA, monounsaturated fatty acids; PUFA, polyunsaturated fatty acids; TFA, total fatty acids. ^1^ Containing (% DM): (i) CTRL, 1st cut hay, 47.9; 2nd cut hay, 23.7; concentrate, 28.4; (ii) HAZ, 1st cut hay, 47.7; 2nd cut hay, 23.6; concentrate, 23.5; hazelnut skin, 5.2. ^2^ Calculated values. ^3^ Composition (%): wheat bran, 28.0; barley grain, 23.0; soybean meal, 12.0; beet pulp, 12.0; soy bean hulls, 10.0; wheat grain, 8.0; sugar molasses, 3.7; calcium carbonate, 1.4; sodium bicarbonate, 1.0; salt, 0.5; vit-min mixture, 0.4 (containing: sodium sulfate, 125 g/kg; zinc oxide, 50 g/kg; ionophore, 30 g/kg; ferrous sulfate, 20 g/kg; sodium bicarbonate, 6 g/kg; copper sulfate, 6 g/kg; vitamin E, 18,000 IU/kg; vitamin A, 3,000,000 IU/kg; vitamin D, 3,750,000 IU/kg; potassium chloride, 140 g/kg; ethylene diamine, 0.5 g/kg; cobalt carbonate, 0.09 g/kg; magnesium oxide, 500 mg/kg; manganese oxide, 36 g/kg; selenium, 0.09 g/kg). ^4^ NSC = 1000 − (NDF + CP + EE + ash).

**Table 2:** Dry matter, main fatty acids, and tocopherols intakes of cows fed the control (CTRL) and hazelnut skin (HAZ) diets ^1^.

	Dietary Treatment		Effects ^2^	
Parameter	CTRL (n = 13)	HAZ (n = 13)	DT	SD
DM intake (kg/head/day)				
Hays	11.81	10.46	***	***
Concentrate	5.30	5.36	- ^3^	- ^3^
Total	17.07	15.81	***	***
FA intake (g/head/day)				
C16:0	60.21	64.17	***	***
C18:0	8.99	11.85	***	***
C18:1 *c*9	64.06	217.56	***	***
C18:2 *c*9*c*12	82.74	93.42	***	***
C18:3 *c*9*c*12*c*15	53.78	47.81	***	***
Σ Other FA ^4^	14.93	17.67	***	***
Σ SFA	78.04	85.07	***	***
Σ MUFA	69.83	226.78	***	***
Σ PUFA	136.90	141.57	***	***
TFA	284.77	453.42	***	***
Tocopherols intake (mg/head/day)				
α-tocopherol	476.17	560.32	***	***
δ-tocopherol	50.04	109.67	***	***

Abbreviations: n, number of samples; DT, dietary treatment; ST, sampling time; DM, dry matter; FA, fatty acids; SFA, saturated fatty acids; MUFA, monounsaturated fatty acids; PUFA, polyunsaturated fatty acids; TFA, total fatty acids. ^1^ Total number of measurements collected for each group equal to 442 (13 cows × 34 sampling days). ^2^ *** *p* ≤ 0.001. The effect of interaction between dietary treatment and sampling date (DT × SD) was not significant; therefore, significance is only presented for main effects. ^3^ During the measurement period, all the offered concentrate was eaten by the cows with no refusals. ^4^ C12:0 + C14:0 + C16:1 *t*3 + C16:1 *c*9 + C18:1 *c*11 + C18:3 *c*6*c*9*c*12 + C20:0 + C20:1 *c*9 + C22:0.

**Table 3:** Milk yield and milk main constituents of cows fed the control (CTRL) and hazelnut skin (HAZ) diets ^1^.

	Dietary Treatment		Effects ^2^	
Parameter	CTRL (n = 13)	HAZ (n = 13)	DT	SD
Milk yield (kg/head × day)	18.14	18.34	ns	ns
ECM ^3^ (kg/head × day)	18.27	18.70	ns	0.08
Milk composition (g/kg)				
Fat	34.99	35.97	ns	0.06
Protein	32.76	31.95	0.08	**
Casein	25.64	25.18	ns	ns
Lactose	47.94	48.40	ns	ns
Component yield (g/head × day)				
Fat	625.62	657.69	ns	*
Protein	588.16	581.81	ns	ns
Casein	460.69	458.60	ns	ns
Lactose	873.94	890.04	ns	ns

Abbreviations: n, number of samples; DT, dietary treatment; SD, sampling date; ECM, energy corrected milk. ^1^ Total number of samples analyzed for each group equal to 52 (13 cows × 4 sampling dates). ^2^ ** *p* ≤ 0.01; * *p* ≤ 0.05; ns, not significant (*p* > 0.05). The effect of the interaction between dietary treatment and sampling date (DT × SD) was not significant; therefore, significance is only presented for main effects. ^3^ ECM = [0.327 × milk (kg/d)] + [12.95 × milk fat (kg/d)] + [7.2 milk protein (kg/d)] [41].

**Table 4:** Saturated and branched-chain fatty acids (g/kg fat) in milk of cows fed the control (CTRL) and hazelnut skin (HAZ) diets ^1^.

	Dietary Treatment		Effects ^2^	
Fatty Acid	CTRL (n = 13)	HAZ (n = 13)	DT	SD
C4:0	25.31	26.96	*	ns
C5:0	0.10	0.08	***	ns
C6:0	19.14	17.77	***	ns
C7:0	0.12	0.06	***	ns
C8:0	10.74	8.91	***	*
C10:0	25.09	17.97	***	**
C12:0	30.31	20.54	***	**
C13:0	0.79	0.46	***	***
C14:0	107.05	84.48	***	**
C16:0	243.98	187.63	***	**
C17:0	4.66	4.16	***	ns
C18:0	67.55	111.00	***	***
C19:0	0.21	0.36	***	***
C20:0	0.98	1.02	ns	ns
C22:0	0.38	0.30	***	ns
C13 *iso*	0.33	0.28	***	***
C13 *anteiso*	0.79	0.52	***	*
C14 *iso*	1.60	1.45	***	ns
C15 *iso*	2.77	2.24	***	***
C15 *anteiso*	5.11	4.08	***	ns
C16 *iso*	3.36	2.83	***	***
C17 *iso*	2.98	2.69	***	ns
C17 *anteiso*	5.06	4.39	***	ns
C18 *iso*	0.08	0.04	***	*
C18 *anteiso*	1.56	1.36	***	ns
Σ SFA	578.57	515.72	***	*
Σ BCFA	23.64	19.86	***	ns
Σ *iso* BCFA	11.13	9.52	***	ns
Σ *anteiso* BCFA	12.52	10.34	***	ns

Abbreviations: n, number of samples; DT, dietary treatment; SD, sampling date; SFA, saturated fatty acids; BCFA, branched chain fatty acids. ^1^ Total number of samples for each group equal to 52 (13 cows × 4 sampling dates). ^2^ *** *p* ≤ 0.001; ** *p* ≤ 0.01; * *p* ≤ 0.05; ns, not significant (*p* > 0.05). The effect of interaction between dietary treatment and sampling date (DT × SD) was not significant; therefore, significance is only presented for the main effects.

**Table 5:** Monounsaturated fatty acids (g/kg fat) in milk of cows fed the control (CTRL) and hazelnut skin (HAZ) diets ^1^.

	Dietary Treatment		Effects ^2^	
Fatty Acid	CTRL (n = 13)	HAZ (n = 13)	DT	SD
C10:1 *c*9	2.98	2.16	***	*
C12:1 *c*9	0.78	0.59	***	ns
C14:1 *t*	0.01	0.02	ns	0.07
C14:1 *c*9 + C15:0	18.52	14.15	***	ns
C16:1 *t*	0.50	0.68	***	***
C16:1 *c*9	7.98	5.91	***	ns
C17:1 *t*	0.39	0.36	*	ns
C18:1 *t*5	0.11	0.55	***	***
C18:1 *t*6–9	2.74	7.20	***	***
C18:1 *t*10–11	11.28	18.04	***	***
C18:1 *t*12–14 *+ c*6–8	3.14	6.94	***	***
C18:1 *c*9	128.65	200.69	***	***
C18:1 *c*11	3.37	4.30	***	***
C18:1 *c*12	1.34	1.56	***	***
C18:1 *c*14 + *t*16	2.19	3.70	***	***
C20:1 *t*	0.20	0.20	ns	0.06
C20:1 *c*9	0.84	0.85	ns	ns
C20:1 *c*11	0.25	0.30	***	***
Σ MUFA	166.75	254.04	***	***
Σ C18:1	152.81	242.98	***	***
Σ C18:1 *t*	19.46	36.43	***	***

Abbreviations: n, number of samples; DT, dietary treatment; SD, sampling date; *c*, *cis*; *t*, *trans*; MUFA, monounsaturated fatty acids. ^1^ Total number of samples for each group equal to 52 (13 cows × 4 sampling dates). ^2^ *** *p* ≤ 0.001; * *p* ≤ 0.05; ns, not significant (*p* > 0.05). The effect of the interaction between dietary treatment and sampling date (DT × SD) was not significant; therefore, significance is only presented for main effects.

**Table 6:** Polyunsaturated fatty acids (g/kg fat) in milk of cows fed the control (CTRL) and hazelnut skin (HAZ) diets ^1^.

	Dietary Treatment		Effects ^2^	
Fatty Acid	CTRL (n = 13)	HAZ (n = 13)	DT	SD
C18:2 *t,t* NMID + *t*9*t*12	0.59	1.00	***	***
C18:2 *c*9*t*13 + *t*8*c*12	0.44	0.48	0.09	***
C18:2 *c*9*t*12	0.72	1.18	***	***
C18:2 *c,c* MID + *t*8*c*13	0.74	1.12	***	**
C18:2 *t*11*c*15	1.70	1.48	***	***
C18:2 *t*9*c*12	0.69	0.58	***	ns
C18:2 *c*9*c*12	9.13	9.96	**	ns
C18:2 *c*9*c*15	0.15	0.13	**	ns
C18:3 *c*6*c*9*c*12	0.07	0.06	**	*
C18:3 *c*9*c*12*c*15	4.81	4.17	***	***
C18:2 *c*9*t*11 + *t*7*c*9 + *t*8*c*10	4.00	5.05	***	***
C18:2 *t*10*c*12	0.08	0.09	0.06	**
C18:2 *t*11*c*13 + *c*9*c*11	0.09	0.10	ns	**
C18:2 *t*9*t*11	0.07	0.06	**	0.09
C20:2 *c,c* n6	0.10	0.09	ns	ns
C20:3 n6	0.29	0.26	*	ns
C20:4 n6	0.44	0.38	***	ns
C20:5 n3	0.27	0.22	***	0.08
C22:5 n3	0.24	0.21	***	**
Σ PUFA	24.62	26.60	***	*
Σ C18:2	5.03	5.96	***	***
Σ C18:2 *t*	9.12	11.13	***	***
Σ CLA	4.25	5.30	***	***
Σ n3	7.17	6.21	***	***
Σ n6	16.50	22.00	***	***
Σ n6/Σ n3	2.34	3.61	***	***

Abbreviations: n, number of samples; DT, dietary treatment; SD, sampling date; *c*, *cis*; *t*, *trans*; NMID, non-methylene interrupted diene; MID, methylene interrupted diene; PUFA, polyunsaturated fatty acids; CLA, conjugated linoleic acids. ^1^ Total number of samples for each group equal to 52 (13 cows × 4 sampling dates). ^2^ *** *p* ≤ 0.001; ** *p* ≤ 0.01; * *p* ≤ 0.05; ns, not significant (*p* > 0.05). The effect of the interaction between dietary treatment and sampling date (DT × SD) was not significant; therefore, significance is only presented for main effects.

**Table 7:** Feed efficiency, human-edible feed conversion efficiency indexes, and net food production of cows fed the control (CTRL) and hazelnut skin (HAZ) diets ^1^.

	Dietary Treatment		Effects ^2^	
Parameter	CTRL (n = 13)	HAZ (n = 13)	DT	SD
Feed efficiency ^3^	1.07	1.19	**	*
heFCE				
CP, g/g edible	0.86	1.04	***	ns
GE, MJ/MJ edible	0.87	1.08	***	ns
NFP				
CP, g/d	−83.33	23.44	***	ns
GE, MJ/d	−8.05	3.96	***	ns
Diet cost (€/head × day)	3.47	3.18	***	***

Abbreviations: DT, dietary treatment; SD, sampling date; heFCE, human-edible feed conversion efficiency; NFP, net food production. ^1^ Total number of samples analyzed for each group equal to 52 (13 cows × 4 sampling dates). ^2^ *** *p* ≤ 0.001; ** *p* ≤ 0.01; * *p* ≤ 0.05; ns, not significant (*p* > 0.05). The effect of the interaction between dietary treatment and sampling date (DT × SD) was not significant; therefore, significance is only presented for main effects. ^3^ Calculated as: ECM (kg)/dry matter intake (kg).
